# The effects of subcurative praziquantel treatment on life‐history traits and trade‐offs in drug‐resistant *Schistosoma mansoni*

**DOI:** 10.1111/eva.12558

**Published:** 2017-11-19

**Authors:** Mafalda Viana, Christina L. Faust, Daniel T. Haydon, Joanne P. Webster, Poppy H. L. Lamberton

**Affiliations:** ^1^ Institute for Biodiversity, Animal Health and Comparative Medicine University of Glasgow Glasgow UK; ^2^ London Centre for Neglected Tropical Disease Research Department of Infectious Disease Epidemiology School of Public Health Imperial College London London UK; ^3^ Centre for Endemic, Emerging and Exotic Diseases The Royal Veterinary College University of London London UK; ^4^ Wellcome Centre for Molecular Parasitology University of Glasgow Glasgow UK

**Keywords:** drug resistance, establishment, fecundity, fitness costs, praziquantel, reproduction, *Schistosoma*, state‐space models, survival

## Abstract

Natural selection acts on all organisms, including parasites, to maximize reproductive fitness. Drug resistance traits are often associated with life‐history costs in the absence of treatment. Schistosomiasis control programmes rely on mass drug administration to reduce human morbidity and mortality. Although hotspots of reduced drug efficacy have been reported, resistance is not widespread. Using Bayesian state‐space models (SSMs) fitted to data from an *in vivo* laboratory system, we tested the hypothesis that the spread of resistant *Schistosoma mansoni* may be limited by life‐history costs not present in susceptible counterparts. *S. mansoni* parasites from a praziquantel‐susceptible (S), a praziquantel‐resistant (R) or a mixed line of originally resistant and susceptible parasites (RS) were exposed to a range of praziquantel doses. Parasite numbers at each life stage were quantified in their molluscan intermediate and murine definitive hosts across four generations, and SSMs were used to estimate key life‐history parameters for each experimental group over time. Model outputs illustrated that parasite adult survival and fecundity in the murine host decreased across all lines, including R, with increasing drug pressure. Trade‐offs between adult survival and fecundity were observed in all untreated lines, and these remained strong in S with praziquantel pressure. In contrast, trade‐offs between adult survival and fecundity were lost under praziquantel pressure in R. As expected, parasite life‐history traits within the molluscan host were complex, but trade‐offs were demonstrated between parasite establishment and cercarial output. The observed trade‐offs between generations within hosts, which were modified by praziquantel treatment in the R line, could limit the spread of R parasites under praziquantel pressure. Whilst such complex life‐history costs may be difficult to detect using standard empirical methods, we demonstrate that SSMs provide robust estimates of life‐history parameters, aiding our understanding of costs and trade‐offs of resistant parasites within this system and beyond.

## 1 INTRODUCTION

Parasite populations often exhibit considerable genetic variability in their natural tolerance, or acquired resistance, to drugs (Pollitt, Sim, Salathe, & Read, 2015). Evolutionary theory suggests that one potential mechanism for maintaining such diversity, particularly when considering resistance, is fitness costs associated with such resistance compared to susceptible lines (Hughes & Andersson, 2015). The existence of life‐history costs and trade‐offs between them is supported by theoretical and empirical studies from both natural and artificial environments across a wide range of organisms (Andersson & Hughes, 2010; Kempf & Zeitouni, 2009; Koella & Antia, 2003; Koella & Zaghloul, 2008). However, convincing demonstrations of costs of drug resistance within parasites and pathogens, and their underlying causes, have proved elusive, particularly in animal host–parasite systems.

Schistosomiasis is a debilitating disease caused by infection with trematode blood flukes. One of the main species affecting humans is *Schistosoma mansoni,* which causes intestinal schistosomiasis across sub‐Saharan Africa and in pockets of South America and the Middle East (GBD 2016). Resistance to praziquantel treatment was first recorded in *S. mansoni* in the mid‐1990s (Ismail et al., 1996, 1999). In 2015, 61 million people were treated during mass drug administration (MDA) campaigns, out of the estimated 218 million people across 52 countries who require treatment (WHO 2016). Despite this scale of drug pressure and a number of reports suggesting reduced praziquantel efficacy in response to MDA (Black et al., 2009; Cioli et al., 2004; Crellen et al., 2016; Danso‐Appiah & De Vlas, 2002; Lawn, Lucas, & Chiodini, 2003; Melman et al., 2009; Wang, Wang, & Liang, 2012), praziquantel resistance still has a limited recorded distribution (Botros et al., 2005; Wang, Dai, Li, Shen, & Liang, 2010; Xu et al., 2015). Whilst observed low cure rates can be caused by a range of factors other than resistance, such as high intensities of infection, high rates of reinfection, low drug absorption and minimal previous immunological exposure to schistosomes, cure rates in some areas are lower than expected despite taking these factors into consideration (Danso‐Appiah & De Vlas, 2002).

A range of potential, not mutually exclusive, explanations have been proposed regarding the apparent lack of emergence and establishment of praziquantel true resistance to date: (i) emergence of resistance may be slow due to obligate dioecious sexual reproduction and a diverse complex life cycle (Botros & Bennett, 2007); (ii) low MDA coverage and untreated life cycle stages outside of the human host provide high levels of *refugia* for susceptible parasites to persist (King, Muchiri, & Ouma, 2000; Park, Haven, Kaplan, & Gandon, 2015); (iii) constraining selective pressures from concurrent snail control (King, Sutherland, & Bertsch, 2015; Xu et al., 2015); or (iv) high costs of resistance that limits their transmission and fixation capability (Leathwick, 2013; William et al., 2001). One early mathematical model indicated that praziquantel resistance could take over a decade to establish due to a combination of these limiting factors (King et al., 2000). Widespread praziquantel resistance would undermine the current schistosomiasis control strategies, as there are currently no alternative accessible drug treatments. Therefore, it is important to gather empirical evidence to support or refute each of these hypotheses, to inform appropriate interventions at a public health level. Such studies also help understand more broadly the complex processes involved in the evolution of resistance in multihost systems.

Here, using a laboratory host–parasite system, we explore the hypothesis that there are costs associated with resistance which may act to limit the spread of praziquantel‐resistant parasites. Whilst some *S. mansoni* isolates can maintain resistance in the laboratory without praziquantel pressure (Cioli et al., 2004), other *S. mansoni* isolates have been reported to revert to susceptibility in as few as six mouse passages without treatment (William et al., 2001). In the absence of drug pressure, praziquantel‐resistant *S. mansoni* lines can have lower cercarial production relative to their susceptible counterparts (William et al., 2001). However, there are inconsistencies across resistant lines, with some lines having lower adult worm establishment and fecundity compared with praziquantel‐susceptible lines (William et al., 2001). Reversion to susceptibility and evidence of costs in definitive hosts for some resistant lines provide support that life‐history costs could act to limit the spread of those resistant lines. However, more experimental studies are needed to explicitly measure these differences between lines and how these vary under selection pressures.

We combined a snail and mouse laboratory study with statistical modelling to elucidate the life‐history traits, and trade‐offs between these traits, across varying doses of *in vivo* praziquantel in drug‐susceptible, drug‐resistant and mixed (50% susceptible and 50% resistant) *S. mansoni* lines across four parasite generations. We test the hypotheses that (i) costs associated with praziquantel resistance, displayed within either the intermediate or definitive host stage, would reduce the overall fitness of the line relative to its susceptible or mixed‐selected counterparts in the absence of drug pressure, and that net costs would be dependent on both (ii) praziquantel dose and (iii) selection generation.

## 2 MATERIALS AND METHODS

### 2.1 Experimental design

**Figure 1:**
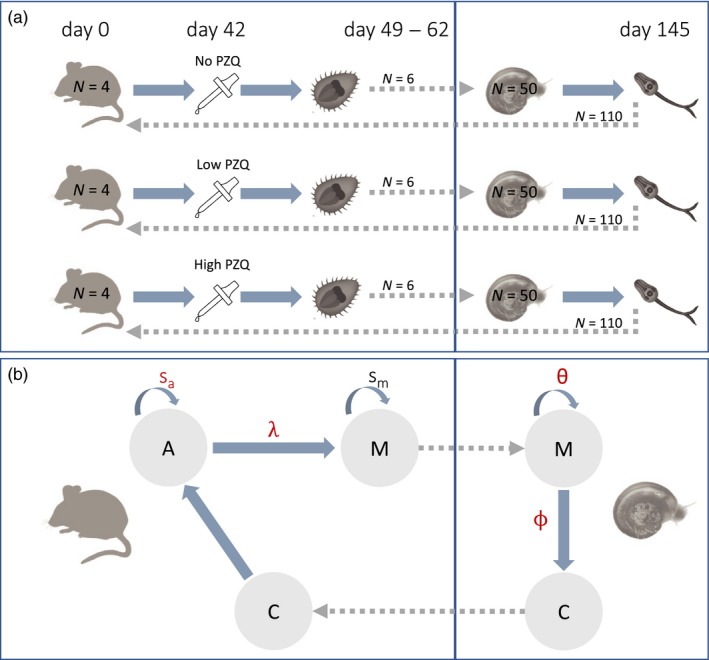
Simplified experimental design and life cycle of *S. mansoni* (as modelled). (a) Each parasite line (S, R and RS) was maintained in the laboratory through passage in four mice and 50 snails in each generation for each praziquantel treatment regime. In generation 1 (G1), mice were infected with 220 cercariae, and in subsequent generations (G2–G4), 110 cercariae were used to infect mice. In all generations, six miracidia were used to infect snails. (b) The life cycle of schistosomes was modelled using particular life stages. Cercariae (C) enter the definitive mouse host and establish themselves as adults (A). Those adults that survive (with survival rate *s*_a_) will sexually reproduce and lay eggs with a rate λ, which hatch into miracidia (M). The miracidia that survive (with a rate *s*_*m*_) will then leave the mouse host and enter the snail. The miracidia (M) establish inside the intermediate snail host with a rate θ and will start shedding sporocysts that become cercariae (C) at a rate ϕ. A new generation will begin as the new cercariae enter the definitive host. The parameters in red correspond to those which can be impacted by praziquantel. The dotted arrows indicate steps that occurred through experimental manipulation. The schistosome images are modified with permission from Genome Research Limited (https://www.yourgenome.org/facts/what-is-schistosomiasis)

For the selection experiment, full details of the experimental methodology are described in Lamberton, Faust, and Webster (2017). Briefly, there were nine treatment groups in a full‐factorial design with three *S. mansoni* parasite lines (praziquantel‐resistant (R), susceptible (S) and mixed infection (RS: 50% R and 50% S in the first generation)) and three praziquantel doses administered to mice (control—sham 2% cremophor EL; low—25 mg/kg praziquantel diluted in 2% cremophor EL; high—50 mg/kg praziquantel in 2% cremophor EL). Both doses of praziquantel were subcurative for all parasite lines to ensure long‐term maintenance of the line. Parasites from each treatment group were maintained for four generations (G1–G4), using four definitive hosts (female Tuc Ordinary (TO) Harlan^®^ adult mice) and 50 indeterminate hosts (30 *Biomphalaria glabrata* and 20 *B. alexandrina*) in each generation (Figure 1a and Appendix Fig. S1).

#### 2.1.1 Observational parasite life‐history data

Mice were exposed to a fixed number of cercariae (220 in G1 and then 110 in G2–G4 due to minor mouse morbidity in G1) from the selection line. 6 weeks (42 days) after exposure to cercariae, mice were orally gavaged with control, low or high doses of praziquantel. Between 49 and 62 days postexposure, mice were euthanized and adult schistosomes counted following collection with a modified hepatic perfusion technique (Smithers & Terry, 1965). An exception occurred at day 42 in G1 when two mice from the low‐dose R treatment arm became ill and had to be euthanized earlier. Counts of adult schistosomes were also collected for these mice. Eggs obtained from the liver and spleen of each mouse were washed in saline as described in Lamberton et al. (2017) and placed in direct light in 70 ml spring water for 1 hr to induce hatching of miracidia. Estimates of viable miracidia were obtained by counting the number of miracidia in ten 0.2‐ml samples per mouse.

**Figure 2:**
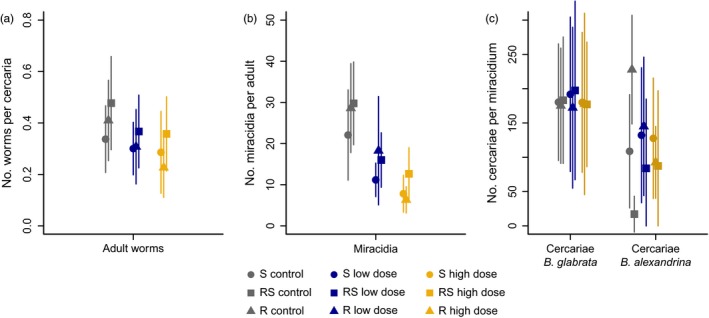
Raw data counts of *Schistosoma mansoni* in the different life cycle stages for each parasite line (symbols: S susceptible; R: resistant; RS: resistant–susceptible) and praziquantel treatment group (colours). Average number (±standard deviation) of (a) adult worms per cercaria and (b) miracidia per adult worm/day and (c) the weekly number of cercariae produced per miracidium (right) are estimated across the four generations for each line/treatment group

To initiate the asexual stage of the schistosome life cycle, 50 *Biomphalaria* snails (30 *B. glabrata* and 20 *B. alexandrina*) per treatment group in 5 ml spring water were each exposed to six miracidia that were pooled from all mice within the same experimental treatment group. Miracidia remaining after 2 hr of exposure were counted and discarded. Snail survival was recorded weekly. Each week, from 3 weeks post‐parasite exposure (to allow for maturation of sporocysts and subsequent cercarial production (Rollinson & Johnston, 1996)), snails were placed in the dark for 24 hr and then exposed to 2 hr of light, at 11 a.m., in glass vials containing 16 ml spring water, to prompt cercarial shedding. The number of cercariae was estimated in two 0.2‐ml aliquots per snail. Due to variation in the number of shedding snails, which may have been influenced by factors external to the experimental treatments, cercariae were pooled from ten snails (including any *B. alexandrina* where possible) at 10 weeks postexposure to improve consistency between treatment groups and generations. These were then used to infect the next generation of mice. This protocol was continued until generation four (G4) (Figure 1a and Appendix S1 Fig.S1). Raw data for this experiment are shown in Figure 2 and Appendix S2 Fig.S2.

### 2.2 Parasite life cycle state‐space model

To understand the complexities governing potential variation in life‐history traits and trade‐offs in *S. mansoni* parasite lines, and how these may be impacted by low and high praziquantel doses, we modelled the life cycle of the three lines of *S. mansoni* across generations. We developed two Bayesian stage‐structured population models implemented within a state‐space modelling (SSM) framework. The first model emulated the sexual portion of the worm life cycle inside the definitive mouse host. The second model recreates the asexual portion of the life cycle inside the intermediate snail host. Figure 1b illustrates the stages of the worm life cycle as they were modelled and how each stage relates to the experimental data described in Figure 1a. Two independent models were implemented as the transition between hosts was experimentally manipulated; that is, there were not natural population dynamics between hosts or generations; for example, each snail was exposed to a fixed six miracidia (indicated by dotted lines in Figure 1). We chose a state‐space model approach because it inherently recognizes that the observation process (as determined here by the experimental design, sampling and data collection protocols) is not a direct mirror of the biological process (the parasite's life cycle) underlying the creation of these observations (e.g., our observations are partial counts of the true miracidia/worm/cercariae numbers) and that the reconstruction of this biological process requires integration of both these processes (Harvey et al. 2004). Furthermore, the ability to use priors based on previous studies strengthens our inference. Finally, similar model structures can be used for both the sexual and asexual component of the life cycle, which facilitates interpretations of the results across the parasites' entire life cycle.

#### 2.2.1 Within‐mouse model

The sexual life cycle model starts with cercariae (C) establishment inside the mouse. After establishment, cercariae develop into adults (A) and the adults that survive can reproduce and lay eggs. Once exposed to freshwater, these eggs hatch into miracidia (M). With the within‐mouse model (Figure 1), we estimate the adult survival (*s*_A_) and fecundity (λ) rates for each parasite line across the four generations and the impact of low and high praziquantel doses on these two traits. Specifically, we model the number of cercariae in replicate *i* from generation *t* that became established inside the mouse and survived to full adult, *n*_*A*,__*i,t*_ as a binomial process parameterized by the number of cercariae each mouse is exposed to (*n*_*A,i,t*_ = 220 in *t* = 1 and *n*_*A,i,t*_ = 110 in *t* = 2,3,4) and probability *s*_*A,i,t*_ describing the combined establishment and adult survival rate (Figure 1), which was expressed as a logit function of low or high drug dose:

(1) $$\text{logit}\left(s_{A,i,t}\right)=\beta_{0,l}-\beta_{1,l,t}{\text{Low}}_{i,t}-\beta_{2,l,t}{\text{High}}_{i,t}$$

The baseline daily survival of each parasite line (*l*) β_0,*l*_ was given a prior distribution derived from a beta distribution with mean 0.85 and variance 0.01 (transformed to the logit space; see derivation in Appendix S3Table S1). The prior is lower than typical daily survival rates to account for low establishment (Smithers & Terry, 1965), but the variance is wide enough to capture values of 1 or near 0. The coefficients β_1,*l,t*_ and β_2*,l,t*_ governing the impact of low and high treatment in each parasite line *l* and generation *t* were estimated using normal distribution priors with mean 0 and precision 0.01; hence, the impact of praziquantel on survival can be positive or negative. The praziquantel doses were modelled as binary switches (i.e., whether a replicate was exposed to high (High_*i,t*_ = 1, Low_*i,t*_ = 0), low (High_*i,t*_ = 0, Low_*i,t*_ = 1) or no praziquantel (High_*i,t*_ = 0, Low_*i,t*_ = 0) dose to facilitate computation of these separate treatments in each parasite line and generation). Hence, here the adult survival rate is a combination of the adult worm survival and the establishment rate of the cercariae inside the mouse, and the baseline β_*0,l*_ refers to the survival of control individuals from each line, that is, those that were not exposed to any treatment in any generation.

The adults then reproduce, and the number of miracidia (*n*_*M0,i,t*_) emerging per replicate *i* and generation *t* is estimated as a function of the fecundity rate λ_*i,t*_ (Figure 1), the number of adult worms *n*_*A,i,t*_ and the adult survival rate *s*_*A,i,t*_:

(2) $$n_{M0,i,t}=\lambda_{i,t}*n_{A,i,t}*\sum_{T=1}^{x}{s_{A,i.t}}^{\Delta T}$$

Similar to the survival rate, the fecundity rate λ_*i,t*_ was also expressed as a function of praziquantel dose:

(3) $$\lambda_{i,t}=\lambda_{0,l}-\lambda_{1,l,t}{\text{Low}}_{i,t}-\lambda_{2,l,t}{\text{High}}_{i,t}$$

where λ_0,*l*_ corresponds to the baseline daily fecundity rate of each line and was modelled with a normal prior distribution with a mean defined as the average number of eggs laid per day (*n* = 365; Loker (1983)) per worm pair, of which 34% mature to miracidia (Chamez, 2006) (mean = 365*0.5*0.34 = 62.05); that is, the fecundity rate is an estimate of the number of eggs laid by one unexposed pair of worms that hatch and become miracidia. The coefficients λ_1,*l,t*_ and λ_2,*l,t*_ governing the impact of low and high treatment were estimated using normal distribution priors with mean 0 and precision 0.01, and praziquantel doses were again modelled as binary switches.

Because the mice were not all sacrificed at the same time, worms that stayed alive longer during the experiment were likely to have laid more eggs during their lifetime. We deal with this potential bias in worm counts by weighting the number of adults *n*_*A,i,t*_ by a summation of survival *s*_*A,i,t*_ across the number of days mature reproducing worms were alive in each replicate Δ*T*, which ranges from *T* = 1, that is day 42 when the first mouse was culled and miracidia counted, to *x* = 20 days, that is day 62 when the last mouse was culled. This allowed to estimate the cumulative fecundity of worms over their mature lifespan in the mouse.

The number of miracidia that survive *n*_*M,i,t*_ was modelled as a binomial process of *n*_*M0,i,t*_ and probability s_*M,i,t*_ describing the miracidia survival rate expressed as a logit function of a parasite line‐specific baseline assuming a prior based on a logit of a beta distribution of mean 0.85 and variance 0.01.

(4) $$n_{M,i,t}∼B\left(n_{M0,i,t},S_{M,i,t}\right)$$

Finally, the number of miracidia in the mouse was estimated based on the observed data by modelling it as a binomial process of the number of miracidia that survived *n*_*M,i,t*_ and the proportion *p* of miracidia in the population that were counted. Given that we have no estimates of the proportion of miracidia we can observe, the parameter *p* was estimated using an uninformative uniform prior distribution ranging from 0 to 1. Similarly, the total number of adults observed (observational data) is modelled as a binomial process of the number adult that survived *n*_*A,i,t*_ and the proportion *q* that are counted. Perfusion techniques enable accurate quantification and identification for schistosomes; hence, the parameter *q* was estimated using a *beta* distribution prior with mean 0.9 and variance 0.01.

#### 2.2.2 Within‐snail model

The asexual stage of the life cycle starts with miracidia establishment inside the snail host. Once established, the miracidia that survive develop into sporocysts which asexually reproduce and grow into cercariae. In the within‐snail model, we estimated the miracidia infectivity and cercarial shedding rates across the four generations (Figure 1) and explore the impact of praziquantel dose in the previous mouse host on the cercarial shedding rate.

The within‐snail model is similar to the within‐mouse model. Specifically, the number of miracidia which established *n*_*Me,i,t*_ inside each snail replicate *i* from generation *t* was modelled as a binomial process parameterized with the number of miracidia the mouse was exposed to (*n* = 6) and the establishment rate θ_*i,t*_. In turn, θ_*i,t*_ is expressed as a logit function of the baseline establishment rate of each parasite line and generation using a prior derived from a *beta* distribution with mean 0.85 and variance 0.01.

Once established, the average weekly number of cercariae *n*_*C0,i,t*_ emerging per replicate *i* and generation *t* is estimated as a function of the cercarial shedding rate ϕ_*i,t*_, and the number of miracidia entering into the snail:

(5) $$n_{C0,i,t}=\phi_{i,t}*n_{M,i,t}$$

The miracidia develop into and shed cercariae with an average weekly rate ϕ_*i,t,*_ which was modelled following equation (3), with the prior for the baseline shedding rate for each line and generation derived from a gamma distribution parameterized with mean 200 and variance 1000. The coefficients governing the impact of low and high treatment were modelled also from a gamma distribution with mean 0.1 and variance 0.01.

Finally, the number of cercariae in the snail *n*_*C,i,t*_ was estimated based on the observed data by modelling it as a binomial process of the number cercariae that survived *n*_*C0,i,t*_ and the proportion *P* of observed cercariae, which is estimated using an uninformative uniform prior distribution ranging from 0 to 1. Appendix S3 Table S1 summarizes the priors used in both models and provides an example of a prior derivation for the logit space.

#### 2.2.3 Model fitting

The model was fitted using the softwares R and JAGS (Plummer, 2003), and two chains were run until full convergence was achieved in all parameters; that is, from 10^6^ iterations run, the first half were discarded and every 100th iteration kept for analysis (thinning). Convergence was assessed through visual inspection of the trace plots as well as using the Heidelberg diagnostic test. Model performance was further assessed through the model fit (i.e., similarity between observations and model estimates; see Appendix S3 FigsS3–S5) and congruence between prior and posterior distributions (posterior distribution should be narrower but fall within the prior distribution).

## 3 RESULTS

The results of the SSMs were used to address our three hypotheses. Firstly, the differences in the life‐history traits of the controls (baselines) estimated for each parasite line (S, RS and R) enable identification of fitness costs and the net fitness associated with praziquantel resistance. Net fitness was estimated within each generation and evaluated as the trade‐off between adult worm survival and fecundity in the mouse host and miracidia establishment and cercarial production in the snail host. Secondly, the comparison of these traits in the control groups with those in the presence of low and high doses of praziquantel shows how fitness is impacted by praziquantel dose administrated to the definitive host. Thirdly, to investigate whether there was selection for resistance under drug pressure, the individual traits within each line and treatment group were compared across generations. Our parasite life cycle models provided a good fit to the data observed (Fig. S2) and converged appropriately, supporting robustness of the model and estimated life‐history parameters.

### 3.1 Life‐history traits but not trade‐offs differ between parasite lines in control groups

In the untreated control groups, the number of observed adult worms, miracidia and cercariae differed between parasite lines (Figure 2). The RS line had the greatest overall fitness with the highest number of worms per cercaria, the highest miracidia per adult worm and the highest cercariae per miracidium in *B. glabrata* snails. In the sympatric *B. alexandrina* snails*,* however, RS had the lowest observed cercarial production. These counts were, nevertheless, variable between generations (Appendix S2 Fig. S2).

**Figure 3:**
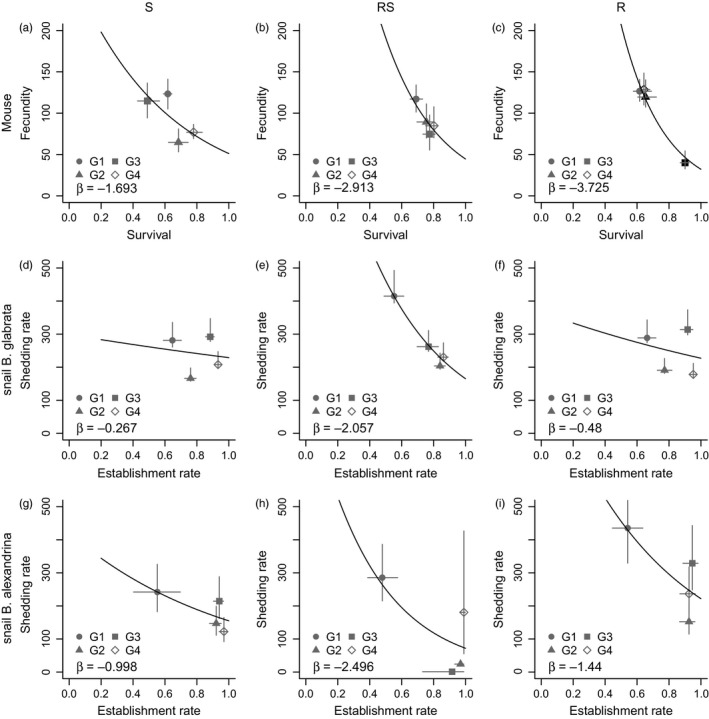
Untreated control (baseline) life‐history traits and trade‐offs in the definitive (a–c) and intermediate (d–i) hosts. For each *Schistosoma mansoni* line, susceptible (S; left), mixed resistant and susceptible (RS; middle) and resistant (R; right), the median (with 95% CIs) life‐history traits (*x*‐ and *y*‐axis) are shown for each generation. The trade‐offs between traits (black line and associated slope, β) were obtained from a Poisson GLM of the posterior distributions of survival (a–c) or establishment (d–i) rates against those of fecundity (a–c) or shedding (d–i) rates, respectively. All regressions were significant with *p*<.001. *p*‐values and standard deviations shown in Appendix S3 TableS2

In the definitive host, the adult survival rate estimated from the life cycle model is a combination of the adult worm survival and the establishment rate of the cercariae inside the mouse host. The average survival of S worms was the most variable across generations, ranging from 0.51 in G3 to 0.80 worms/cercaria in G4, and was generally lower compared to other lines (Table 1; Figure 3a–c, *x*‐axis). For example, the average survival across generations in S line was 0.65 and in R line was 0.71. RS parasites had the most similar rates of adult worm survival between generations, with an average of 0.77 worms/cercaria (Figure 3b). The estimated fecundity rate for control groups was highly variable across our experimental conditions, ranging from 40 (R, G3) to 127 (R, G1) viable miracidia/worm/day depending on generation and parasite line (Table 1; Figure 3a–c, *y*‐axis). However, the fecundity rate of control R worms was generally higher, with a mean across generations of 104 miracidia/worm/day, compared to 95 and 91 miracidia/worm/day in S and RS lines, respectively. Negative trade‐offs between survival and fecundity rates were estimated for all control groups among all parasites lines, with fecundity decreasing with increasing adult worm survival (Figure 3a–c, black line).

**Table 1:** SSM estimated key sexual life‐history parameters. Median (95% CIs) adult survival and fecundity rates baselines (i.e., control groups) and the percentage of reduction over that baseline caused by low and high doses of praziquantel in each generation and line

		Susceptible	Resistant–Susceptible	Resistant
Survival				
Baseline (control)	G1	0.62 (0.59–0.75)	0.71 (0.66–0.80)	0.65 (0.57–0.86)
	G2	0.70 (0.62–0.79)	0.77 (0.70–0.91)	0.66 (0.60–0.84)
	G3	0.51 (0.40–0.71)	0.78 (0.74–0.87)	0.89 (0.85–0.93)
	G4	0.80 (0.74–0.90)	0.81 (0.77–0.90)	0.65 (0.61–0.74)
% Reduction low praziquantel	G1	29.86 (22.7–37.3)	38.97 (33.0–45.1)	30.09 (19.1–42.3)
	G2	25.33 (15.4–35.8)	19.86 (13.1–26.5)	28.86 (20.2–38.2)
	G3	9.05 (0.0–20.1)	23.01 (16.9–28.9)	44.58 (33.5–54.0)
	G4	21.10 (13.4‐29.0)	9.65 (0.0–19.5)	0 (0–1.1)
% Reduction high praziquantel	G1	53.24 (44.0–59.3)	8.88 (0.4–17.5)	52.39 (42.2–59.3)
	G2	30.63 (17.6–39.7)	31.74 (23.2–39.4)	48.84 (37.5–57.6)
	G3	8.17 (0.0–19.7)	22.69 (16.1–29.7)	49.19 (42.1–55.7)
	G4	12.54 (2.3–23.0)	19.43 (12.6–25.2)	40.48 (32.5–47.7)
Fecundity				
Baseline (control)	G1	123 (105–141)	117 (101–134)	127 (114–141)
	G2	65 (53–81)	89 (70–111)	120 (107–140)
	G3	115 (94–136)	75 (55–98)	40 (33–54)
	G4	77 (69–86)	85 (70–108)	129 (110–149)
% Reduction low praziquantel	G1	27.69 (17.97–36.81)	0.0 (0.0–0.0)	0.0 (0.0–5.38)
	G2	0.0 (0.0–8.59)	2.97 (0.0–17.05)	4.34 (0.0–16.20)
	G3	0.0 (0.0–5.99)	0.0 (0.0–0.0)	0.0 (0.0–0.0)
	G4	31.02 (13.81–46.57)	40.76 (27.74–53.53)	0.0 (0.0–8.41)
% Reduction high praziquantel	G1	4.98 (0.0–14.11)	42.30 (31.13–50.78)	14.58 (2.28–23.03)
	G2	44.21 (26.83–58.00)	21.79 (2.46–36.21)	10.56 (0.0–19.11)
	G3	3.78 (0.0–17.11)	21.35 (9.84–34.56)	37.29 (24.45–50.80)
	G4	71.07 (60.65–76.95)	17.06 (1.59–35.31)	14.30 (1.80–24.93)

The results from the intermediate host model (Figure 3d–i) show that the establishment rates of miracidia in the untreated controls were highly variable, but similar among parasite lines and between snail host species, ranging between 0.5 and 1 (Figure 3d–i, *x*‐axis). In contrast, the estimated weekly cercarial shedding rates in the untreated control groups differed greatly between the two snail species (Figure 3d–i, *y*‐axis), although no pattern across generation or parasite line was identified. The overall cercariae shedding rate was lower and less consistent in *B. alexandrina* compared with *B. glabrata* (e.g., in RS *B. alexandrina* ~0–300; *B. glabrata* ~200–400 cercariae). However, the data obtained in *B. alexandrina* were highly variable across generations and lines, as large numbers of snails did not survive once patent, which may have interfered with the estimation of the shedding rates. Regardless, in *B. alexandrina*, our results demonstrated a clear trade‐off between establishment and shedding rates, where higher establishment rates led to lower cercariae output (Figure 3g–i, black lines). However, these trade‐offs were not strong for either R or S lines when infecting *B. glabrata* (Figure 3d, f, black lines).

### 3.2 Praziquantel dose‐dependent changes in life‐history traits and trade‐offs in definitive hosts

In the definitive host, the observed data from the experiments demonstrated that treatment with praziquantel lowers the number of adult worms/cercaria and miracidia/adult worm across the three *S. mansoni* lines (Figure 2a,b). The data from the snail experiments demonstrated that praziquantel treatment in the previous mouse host did not impact the average number of cercariae/miracidium neither in snail species (Figure 2c) nor across generations (Appendix S2 Fig.S2). Our models confirmed these observations. Specifically, the within‐snail model estimated a negligible impact of praziquantel on cercarial production. It is possible that praziquantel impacted cercarial output in some line:generation combinations; however, the improved model fit in the absence of these effects, and the inconsistency of its magnitude across the replicates, does not provide enough evidence to conclude that praziquantel treatment in the mouse host can affect cercarial output in the current snail host. Because the effects of praziquantel exposure in the sexual life cycle stage do not appear to carry through to the asexual life cycle stage, this suggests that *S. mansoni* parasites are not impacted by indirect exposure to praziquantel. In this section, we therefore focus on the impacts of praziquantel dose on the sexual life‐history parameters of schistosomes in the definitive mouse host.

**Figure 4:**
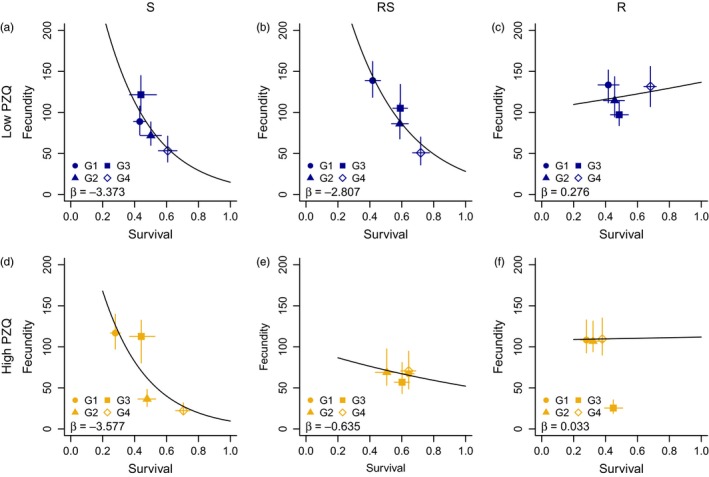
Sexual life‐history traits and trade‐offs under dose‐dependent praziquantel pressure. For each *S. mansoni* line, susceptible (S; left), mixed resistant and susceptible (RS; middle) and resistant (R; right), and praziquantel (PZQ) dose (rows), the median (with 95% CIs) survival (*x*‐axis) and fecundity (*y*‐axis) rates are shown for each generation. The trade‐offs between these traits (black line and associated slope, β) were obtained from a Poisson GLM of the posterior distributions of survival and fecundity. All regressions were significant with *p*<.001, except R low and R and RS high PZQ. *p*‐values and standard deviations shown in Appendix S3Table S2

The results from the within‐mouse model suggest that S lines were more impacted, in terms of fecundity reduction, by praziquantel than R or RS lines (Figure 4 compared to Figure 3a–c; Table 1). Indeed, fecundity, and to a lesser extent survival, was consistently impacted by 50 mg/kg praziquantel although with varying magnitudes across generations (Table 1). Trade‐offs between survival and fecundity in S‐treated lines were similar to control untreated lines, with fecundity decreasing with increasing adult worm survival. In R lines, adult survival was most negatively impacted by 50 mg/kg praziquantel compared to other lines, but fecundity was least affected at the same dosage in nearly every generation (Table 1). The net impact of praziquantel on these traits led to a lack of clear trade‐offs between fecundity and survival (Figure 3c,f), suggesting the life‐history strategies are changed in these R lines even in the presence of low praziquantel doses. Interestingly, in the RS line this change in trade‐offs only occurs in the presence of the higher 50mg/kg praziquantel dose (Figure 4e). This is consistent with its anticipated lower level of resistance compared with the R line, but higher level of resistance compared with the S line (Figure 4).

### 3.3 Evidence of selection during the experiment

Our within‐mouse model showed that praziquantel treatment lowered worm density by impacting both adult survival and fecundity in all parasite lines (Table 1 and Figure 4). However, the lack of decrease with continued praziquantel dose across generations suggests that resistance across generations was not further selected for. Our within‐snail model shows there was positive selection for miracidia infectivity in *B. glabrata,* as seen by the increasing establishment rate of miracidia of all lines across generations: from 0.6 in G1 to ~1.0 in G4 (Figure 3d–f, *x*‐axis). In *B. alexandrina*, the establishment rate was low in G1 but was equally high (near 1) in the following three generations.

## 4 DISCUSSION

**Figure 5:**
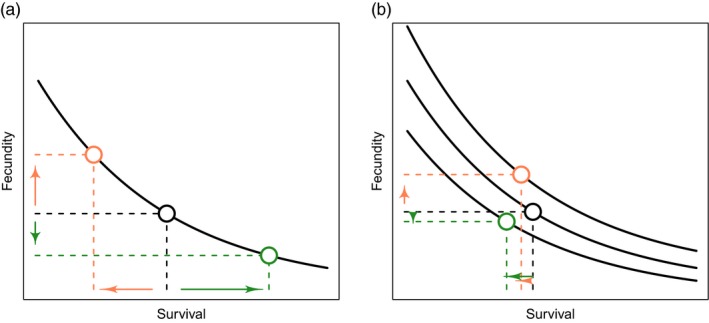
Life‐history traits quantify an organism's investment into components of fitness, such as reproduction (*y*‐axis) and survivorship (*x*‐axis). (a) There are recognized trade‐offs between life‐history traits (solid curve), so that an increase in fecundity is associated with decreased survivorship (orange) or conversely higher survivorship leads to lower fecundity (green). These life‐history trade‐offs arise from limited resources available for an organism's growth, reproduction and survivorship. (b) However, trade‐off curves can shift as resource (energy) availability for certain traits change—this may be due to other resource allocation needs in the organism or a decline in resource availability. When this occurs, negative trade‐offs are difficult to observe and a decline in one trait (orange) may not be compensated by a second observed trait (green). [Figure adapted from work by Noordwijk and de Jong (1986)]

Understanding changes in life‐history traits and potential costs of drug resistance in parasites is important for managing the emergence of resistance and implementing suitable control measures in regions where resistance is found. In this study, we use state‐space models (SSMs) to quantify these costs using empirical data from a laboratory study of an indirectly transmitted parasite. We show that it is possible to measure differences in life‐history traits in parasite susceptible and resistant lines, providing a proof of concept for implementing these techniques in other parasites with complex life cycles. Whilst we observed a large variation in *S. mansoni* life‐history traits between praziquantel‐treated groups and across generations, the parameter estimates generated from the model enabled us to show that variation between groups can be better understood when shown as life‐history trade‐offs. Higher fecundity in untreated parasites was associated with lower survival within the same generations, meaning that net fitness was similar to untreated parasites in another generation that had lower fecundity but higher survival in the definitive host of the same line (Figure 5a). However, when adult worms were exposed to praziquantel, trade‐offs between fecundity and survival in low and high treated R groups and high treated RS groups were no longer observed. This suggests that R, and to some extent RS, respond to drug pressure by altering resource allocation, affecting observations of life‐history trade‐offs (Figure 5b). Whilst this does not explicitly provide support for the hypothesis that costs of resistance limit the spread of praziquantel‐resistant phenotypes in natural populations, it does suggest that life histories are altered in R parasites when exposed to praziquantel, but not altered in the S parasite lines.

Using a *S. mansoni* model, our findings indicate that measuring within‐host parasite life‐history trade‐offs captures better the complexity of host–parasite interactions which are affected by praziquantel, than empirical measures of individual life‐history traits. In comparison with classical statistical methods (Lamberton et al., 2017), the SSM was able to differentiate life‐history traits between parasite lines. This is likely because this SSM method explicitly incorporates the observation process (i.e., experimental design) that generated the data. In contrast to our hypothesis, in the absence of praziquantel, R lines did not exhibit a consistent cost compared to S lines in either host. Although lower adult survival was observed in R lines, this was accompanied by higher fecundity, whilst the negative trade‐off between adult worm survival and fecundity was seen across all three parasite lines in the absence of praziquantel treatment. In the intermediate host, there were also no differences between parasite lines. Life‐history traits of other *S. mansoni* praziquantel‐resistant lines have been also been shown to be similar to praziquantel‐susceptible lines in other laboratory studies (e.g., Cioli and Pica‐Mattoccia (2003) and others). These findings suggest that there are no consistent, observable costs to individual life‐history traits measured here in the absence of treatment.

However, in the presence of drug treatment, R, and to some extent RS, had altered life‐history strategies within the definitive host. A negative trade‐off between survival and fecundity was less apparent in R lines treated with both the low and high praziquantel and RS lines treated with the high dose. Trade‐offs are less likely to be found when resource availability and/or allocation is variable (Noordwijk & de Jong, 1986) (Figure 5b). Drug treatment could modify resource allocation (Johnson et al., 2007), so that R parasites are not only investing energy in survival and reproduction. For RS parasites, a negative trade‐off between fecundity and survival in adult worms was still observed when treated with lower doses of praziquantel (25 mg/kg). However, this trade‐off was altered in the RS line when the higher (50 mg/kg) praziquantel dose was administered, with adult worm fecundity barely decreasing at higher adult worm survival. This latter effect appeared to be similar to the effect for both praziquantel doses in the R line, indicating a complex interaction between drug susceptibility, praziquantel dose and resource allocation. There was no significant difference in adult worm sizes between lines (Lamberton et al., 2017), suggesting that resource allocation towards growth could not explain the absence of negative trade‐offs under drug pressure.

Praziquantel's exact mechanism of action is unknown, but several studies indicate it may be related to calcium (Ca^2+^) channels and tegument damage (Greenberg, 2013). Juvenile worms are not killed by praziquantel, and it is only after they reach sexual maturity that they are praziquantel‐susceptible (Cioli & Pica‐Mattoccia, 2003). A study has shown that juvenile worms increase transcription of stress response genes that may counteract Ca^2+^ influx (Hines‐Kay et al. 2012), potentially explaining their tolerance. If praziquantel induced a similar level of stress in adult worms in R lines, then a similar stress response may be predicted. The loss of a trade‐off between adult worm survival and fecundity in R and RS lines under praziquantel pressure may indicate that R parasites still have the ability to increase transcription of stress response genes to protect against praziquantel and that this additional energy sink may cloud trade‐offs between fecundity and survival.

Drug pressure has increased dramatically since just 2003 (Webster, Molyneux, Hotez, & Fenwick, 2014) but is still currently relatively rare in the field, with each person normally treated once a year or less. Therefore, an alternative explanation for why resistant parasites have not yet become more widespread is that although R parasite lines will be able to establish within a human, their lower overall fecundity makes it less likely that “R” genes would be transmitted onwards. The existence of such trade‐offs could be critical to how resistance spreads, particularly given WHO's recent shift from morbidity control to elimination as a public health problem (WHO 2013) and the planned increase in treatment coverage, frequency and dose (moving to more community‐wide treatment rather than school‐aged children, twice‐a‐year treatments, at potentially a 60 mg/kg dose). Further research is required to explore the range of trade‐off responses and switches to establish whether these are related to different expression profiles (Greenberg, 2013), stress response genes (Hines‐Kay et al. 2012) or other genetic or epigenetic factors.

Previous work has documented lower cercarial production in several resistant lines (William et al., 2001). Although the R and S parasite lines used have different susceptibilities to praziquantel in the definitive mouse host *in vivo*, definitive host life‐history traits were not correlated with traits in the intermediate host, with no differences observed between parasite lines or with praziquantel treatment. This was consistent with our observation that direct exposure to praziquantel has a stronger influence on life‐history parameters than parasite line alone, indicating that we may be reporting phenotypic or inducible effects, rather than simply genotypic selection. Our model did nevertheless support a trade‐off between miracidia infectivity and establishment rate with mean cercarial shedding, similar to that observed in the definitive host. Establishment increased across the experiments, reaching nearly 1.0 in G2 *B. alexandrina*. The increase in establishment rates in both snail species may reflect local adaptation to the particular laboratory strains. Faster adaptation within *B. alexandrina* than in *B. glabrata* may reflect its role as their natural host even in laboratory conditions. Additionally, the larger variation in estimates associated with *B. alexandrina* may be due to a larger genetic variability within the host species compared with the laboratory‐adapted *B. glabrata*.

In the snail host, there were few differences observed between lines and no effect of indirect praziquantel treatment on parasite life histories. Cercarial production was not different between R and S parasite lines; however in early generations of RS, cercarial production was higher than S and R individually. Overall, the mixed RS lines also demonstrated the highest establishment and survival across all generations, relative to their single‐line counterparts, supporting previous findings (Lamberton et al., 2017). In a natural system, a mixed infection may be more likely to result in increased in fitness, if random mating occurs. As *Schistosoma* species rapidly lose genetic variation when passaged in the laboratory (Gower et al., 2007; Stohler, Curtis, & Minchella, 2004), the increased fitness of RS may be explained by an outbreeding effect, through increased genetic diversity, of these two potentially bottlenecked inbred laboratory S and R strains, particularly in the first generation. Some of the variability in the traits measured in these RS lines could also be explained by nonrandom mating and facilitation and/or competition between R and S, or a complex combination of all of these. In the trade‐off analyses discussed above, survival and fecundity trade‐offs observed in the co‐infected RS line with increased treatment fall between R and S lines, correlating with a mid‐level of drug resistance.

Although we used subcurative doses of praziquantel (25 mg/kg and 50 mg/kg in a rodent model system), this level of treatment may be more relevant to actual MDA campaigns than previously thought. We deliberately chose subcurative doses, to enable us to continue the life cycle in the treated selected lines, as this would not have been possible if all S parasites had died with treatment. Cure rates for human treatment are not 100% (Doenhoff, Cioli, & Utzinger, 2008), and therefore, our scenario where some worms survive treatment was aimed to be representative of natural systems. Indeed, there is recent evidence that underdosing may be widespread in children due to pharmacokinetic differences with adults on whom the drug was initially trialled (Bustinduy et al., 2016; Olliaro, Delgado‐Romero, & Keiser, 2014). As the majority of national treatments are administered to children (WHO 2016), modifications to life‐history trade‐offs seen in our experiment may also appear in subcurative treatments in humans. Declines in fecundity estimated here strongly support other empirical studies (Lamberton, Kabatereine, Oguttu, Fenwick, & Webster, 2014), but whether trade‐offs between fecundity and survival are modified in resistant parasites in natural populations remains an open question.

Whilst the experiment was designed to investigate trade‐offs that would be nearly impossible to observe in the field, there were a few limitations of the design. We saw no differences in definitive hosts across generations but this may be due to the lower sample sizes (*n* = 4) for each treatment group per generation, in comparison with the larger sample sizes for snail hosts (*n* = 50 per treatment group). In addition, the R and S parasite lines have been passaged in the laboratory for over a decade and may have lost significant genetic diversity. Although the two strains that were used (MOC‐S and EE2‐R) have significantly different susceptibilities to praziquantel and the levels of susceptibility are stable over laboratory passages even in the absence of drug treatment (Sabra & Botros, 2008; William et al., 2001), potential costs may have been reduced under previous laboratory selection. In addition, although EE2‐R's stability is important to compare between a susceptible and resistant parasite, it might be that the stability is inherently linked to not having high costs of resistance. For example, those which lost their resistance rapidly in the laboratory, or could not be successfully isolated in the laboratory, may have had higher costs initially, minimizing our ability to detect them. Our models, however, did detect subtle differences in the trade‐offs between individual traits and how these changed with treatment, particularly in the R lines, which were not detected using statistical methods alone (Lamberton et al., 2017).

## 5 CONCLUSIONS

Few empirical data are available on the costs of drug resistance. Here, we demonstrate the complexity of quantifying costs and the importance of monitoring not just simple trait‐specific life histories, but within and between host trade‐offs. We observed the expected praziquantel dose‐dependent reduction in *S. mansoni* adult worm survival and establishment in all parasite lines. In addition to the survival and fecundity effects, there was evidence of important changes in life‐history trade‐offs with praziquantel treatment in R lines. The shape of the trade‐off between fecundity and survival in R and S adult worms suggested divergent strategies with increasing *in vivo* drug pressure. These results highlight the importance of monitoring multiple life‐history traits and the trade‐offs between them as well as the complexity in interpreting them. Our study also highlights the complexity of life‐history traits within and between life cycle stages and experimental design, for monitoring evolutionary processes even within a controlled laboratory environment. Mass drug administration against schistosomiasis at the current scale and frequency raises concerns about the emergence of drug resistance in the parasites (Bergquist, Utzinger, & Keiser, 2017; Botros & Bennett, 2007; Webster et al., 2014), but such detailed studies would be impossible to achieve in the field under natural (human definitive host) conditions. Our results thereby provide important insights and have theoretical and applied implications and applications for future schistosomiasis control programmes and for other host–parasite treatment programmes in general.

## DATA ARCHIVING STATEMENT

Data available from the Dryad Digital Repository: https://doi.org/10.5061/dryad.gb682

## AUTHORS' CONTRIBUTIONS

PHLL and JPW designed the project; PHLL undertook the experiments; MV wrote the model and performed the model analyses; DTH helped with model analyses; MV and CLF performed all other the analyses and drafted the initial manuscript; MV, CLF, DTH, JPW and PHLL wrote the final manuscript; and all authors approved the final version of the manuscript.
