# Supplementary material for: The effects of subcurative praziquantel treatment on life‐history traits and trade‐offs in drug‐resistant Schistosoma mansoni
Source: Evol Appl. 2017 Nov 19;11(4):488–500. doi: 10.1111/eva.12558 (PMC5891057; doi:10.1111/eva.12558)
Supplement: Supplementary file 1 [file EVA-11-488-s001.docx]

**APPENDIX**

**The effects of sub-curative doses of praziquantel on survival and reproduction trade-offs amongst drug-resistant *Schistosoma mansoni***

**A. Experimental design**

**Figure S1** – Experimental design adapted from Lamberton, Faust and Webster 2017. In generation 1, mice were infected with 220 cercariae. In generations 2-4, mice were infected with 110 cercariae.

**B. Data**

**Figure S2**. – Observational data obtained from each stage of the *S. mansoni* life cycle during the experiments. Top panels show data from the stages inside the mouse: strain specific (different symbols) mean number of adult worms (left) and miracidia (right) per cercariae, collected in each treatment group (colour) across generations (x-axis). Bottom panels show data from the stages inside the snail: strain specific (different symbols) weekly number of cercariae collected in each treatment group (colour) across generations in each snail species: *B. glabrata* (left) and *B. alexandrina* (right).

**C. Statistical methods and model fit**

**Table S1** – Prior distributions table for all parameters used in the state-space model. We note that the baselines are shown in non-transformed space. See below for an example of the prior derivation for the logit space.

| **Parameter** | **Parameter definition** | **Prior distribution** |
| --- | --- | --- |
| *Within mouse model* | | |
| β_0,l_ | Baseline adult worm daily survival | Beta (µ=0.85, $\sigma^{2}$=0.01) |
| β_1,l,t_ | Impact of low PZQ dose on daily survival | Normal (µ=0, $1/{\sigma^{2}}$=0.01) |
| β_2,l,t_ | Impact of high PZQ dose on daily survival | Normal (µ=0, $1/{\sigma^{2}}$=0.01) |
| λ_0,l_ | Baseline daily fecundity rate | Gamma (µ=62, $\sigma^{2}$=400) |
| λ_1,l,t_ | Impact of low PZQ dose on daily fecundity | Normal (µ=0, $1/{\sigma^{2}}$=0.01) |
| λ_2,l,t_ | Impact of high PZQ dose on daily fecundity | Normal (µ=0, $1/{\sigma^{2}}$=0.01) |
| s_m_ | Miracidia daily survival rate | Beta (µ=0.85, $\sigma^{2}$=0.01) |
| p | Proportion of observed miracidia | Uniform (0,1) |
| q | Proportion of observed adult worms | Beta (µ=0.9, $\sigma^{2}$=0.01) |
| *Within snail model* | | |
| θ_0,l_ | Baseline miracidia establishment rate | Beta (µ=0.85, $\sigma^{2}$=0.01) |
| _0,l,t_ | Baseline shedding rate | Gamma (µ=200, $\sigma^{2}$=1000) |
| _1,l,t_ | Impact of low PZQ dose on shedding | Normal (µ=0, $1/{\sigma^{2}}$=0.01) |
| _2,l,t_ | Impact of high PZQ dose on shedding | Normal (µ=0, $1/{\sigma^{2}}$=0.01) |
| P | Proportion of observed cercariae | Uniform (0,1) |

Example of the derivation for the baseline adult daily survival prior. We need a prior with mean=0.85 and variance (var)=0.01. A beta distribution is parameterized in terms two shape parameters a and b. The derivation of the shape parameters from the mean and variance is as follow:

$$a=\frac{mean}{var}*(mean- \mathrm{mean}^{2}-var)$$

$$b=\frac{1-mean}{var}*(mean- \mathrm{mean}^{2}-var)$$

The value of the prior β_0_ is then defined as the logit of a draw *x* from the beta distribution:

$$x\sim Beta(a,b)$$

$${}_{0}=log(\frac{x}{1-x})$$

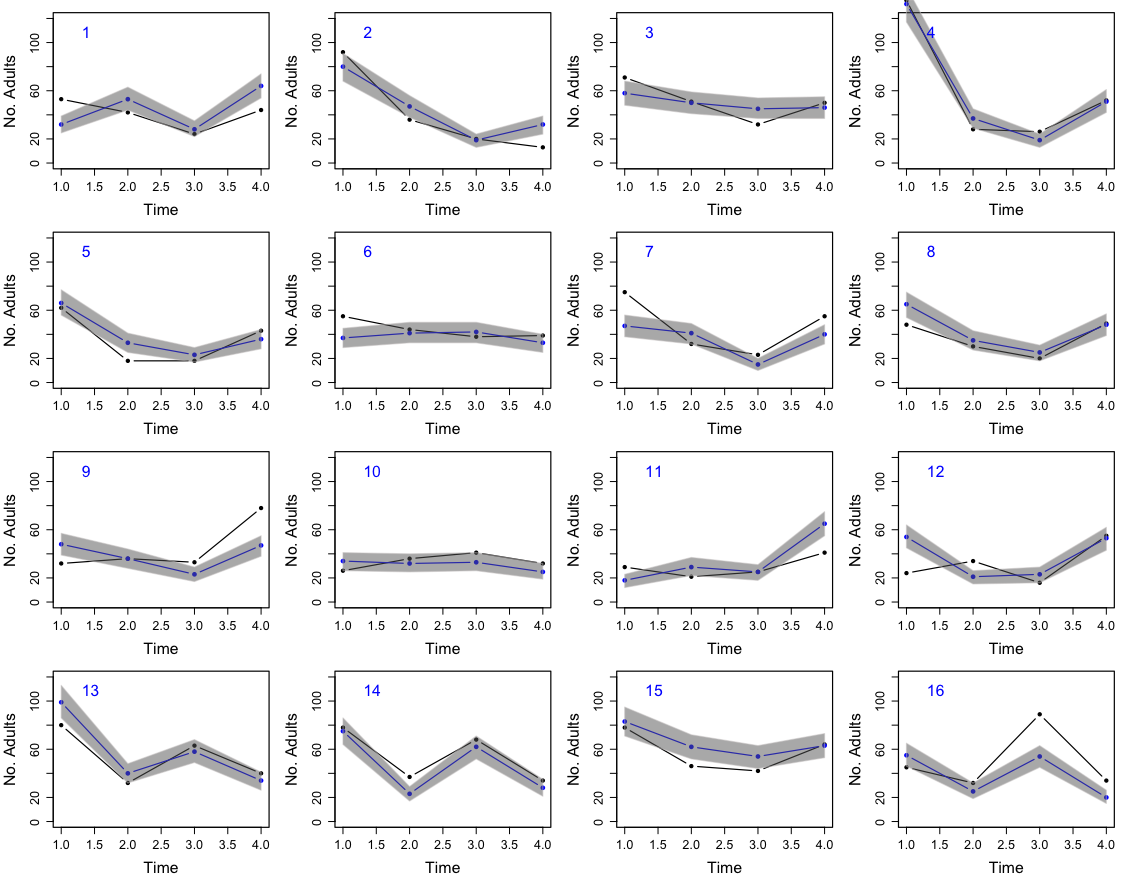


**Figure S3**. Example of model fit to the data of adult worms in sixteen different mice. Black lines correspond to the data and blue line with grey 95% credible intervals correspond to the model estimates.


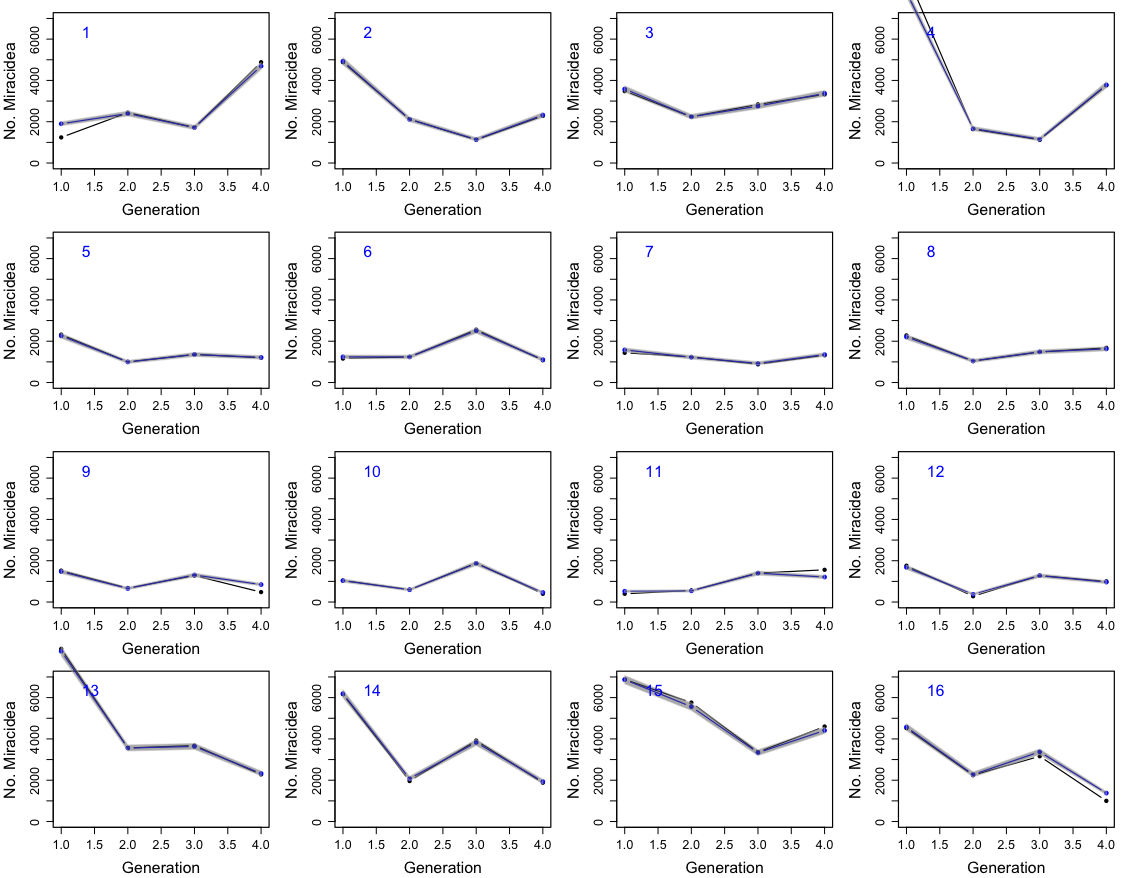


**Figure S4**. Example of model fit to the data of miracidia in sixteen different mice. Black lines correspond to the data and blue line with grey 95% credible intervals correspond to the model estimates.


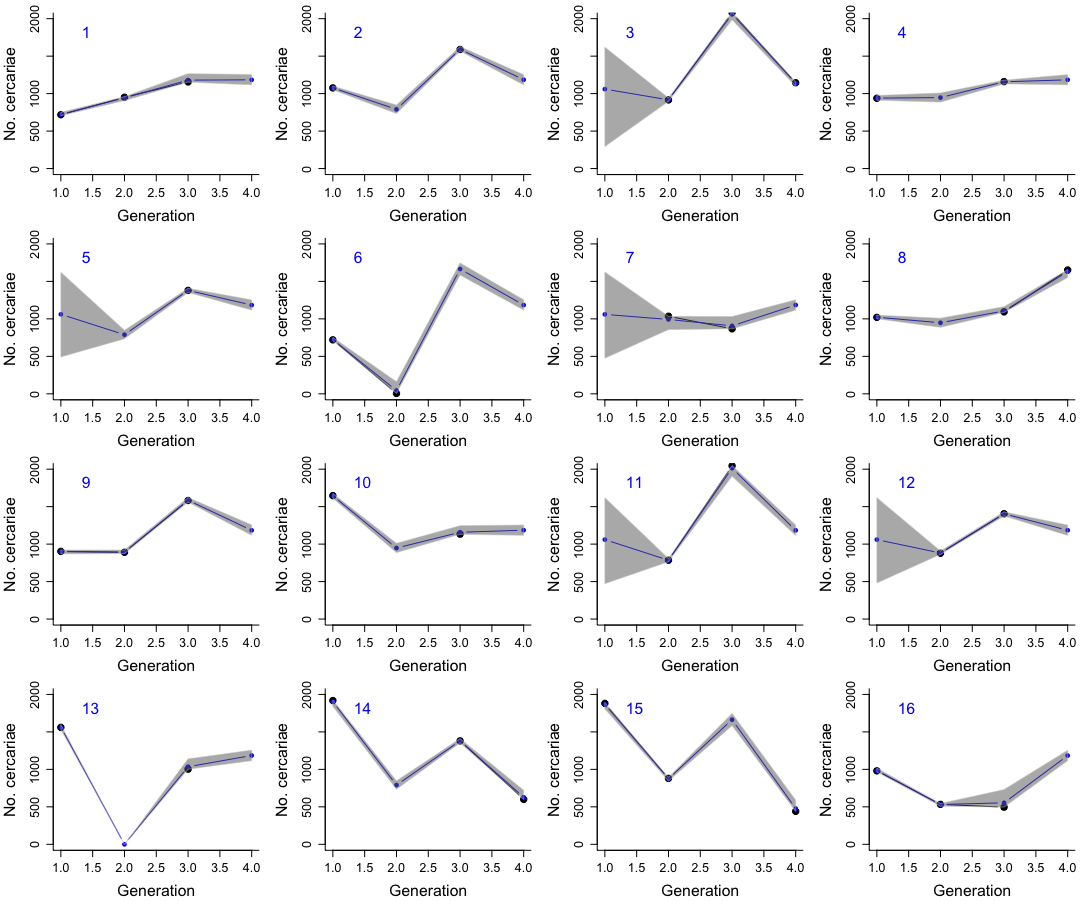


**Figure S5**. Example of model fit to the data of cercariae in sixteen different snails. Black lines correspond to the data and blue line with grey 95% credible intervals correspond to the model estimates.

**Table S2** – Coefficient values obtained from the poisson GLM used to access life-history trade-offs in Figures 3 and 4 of the main manuscript. PZQ=praziquantel.

| **Treatment group** | **S** | **RS** | **R** |
| --- | --- | --- | --- |
| ***Survival vs fecundity*** | | | |
| **Baseline**  *Intercept:* Est. ± sd  Pr(>\|z\|)  *Slope (β):* Est. ± sd  Pr(>\|z\|) | 5.63±0.004  <2e-16  -1.69±0.006  <2e-16 | 6.71±0.01  <2e-16  -2.91±0.016  <2e-16 | 7.20±0.005  <2e-16  -3.72±0.007  <2e-16 |
| **Low PZQ**  *Intercept:* Est. ± sd  Pr(>\|z\|)  *Slope (β):* Est. ± sd  Pr(>\|z\|) | 6.07±0.005  <2e-16  -3.37±0.010  <2e-16 | 6.14±0.004  <2e-16  -2.81±0.006  <2e-16 | 4.64±0.003  <2e-16  0.28±0.006  <2e-16 |
| **High PZQ**  *Intercept:* Est. ± sd  Pr(>\|z\|)  *Slope (β):* Est. ± sd  Pr(>\|z\|) | 5.84±0.003  <2e-16  -3.58±0.006  <2e-16 | 4.58±0.008  <2e-16  -0.63±0.014  <2e-16 | 4.68±0.003  <2e-16  0.03±0.010  0.0007 |
| ***Establishment vs shedding*** | | | |
| **Baseline B. glabrata**  *Intercept:* Est. ± sd  Pr(>\|z\|)  *Slope (β):* Est. ± sd  Pr(>\|z\|) | 5.70±0.003  <2e-16  -0.27±0.004  <2e-16 | 7.16±0.002  <2e-16  -2.06±0.003  <2e-16 | 5.90±0.003  <2e-16  -0.48±0.003  <2e-16 |
| **Baseline B. alexandrina**  *Intercept:* Est. ± sd  Pr(>\|z\|)  *Slope (β):* Est. ± sd  Pr(>\|z\|) | 6.04±0.002  <2e-16  -1.00±0.002  <2e-16 | 6.77±0.001  <2e-16  -2.50±0.002  <2e-16 | 6.84±0.002  <2e-16  -1.44±0.002  <2e-16 |
